# 1888. Implementation of COVID-19 Surveillance Testing Procedures in Dental School

**DOI:** 10.1093/ofid/ofac492.1515

**Published:** 2022-12-15

**Authors:** Holly Shoemaker, Jeri Bullock, Kristina Stratford, Melodie L Weller, Wendy L Hobson-Rohrer, Wendy L Hobson-Rohrer, Matthew H Samore, Jeanmarie Mayer, Lindsay D Visnovsky

**Affiliations:** University of Utah, Salt Lake City, Utah; University of Utah, Salt Lake City, Utah; University of Utah, Salt Lake City, Utah; University of Utah, School of Dentistry, Salt Lake City, Utah; University of Utah, Salt Lake City, Utah; University of Utah, Salt Lake City, Utah; University of Utah, Salt Lake City, Utah; University of Utah, Salt Lake City, Utah; University of Utah School of Medicine, Salt Lake City, Utah

## Abstract

**Background:**

Dental practitioners and students of dentistry are potentially at increased risk of COVID-19 infection due to frequent usage of aerosol-generating procedures. To mitigate risk to patients and providers, the University of Utah School of Dentistry began regular surveillance PCR testing of its patient-facing faculty, staff, and students in May 2020.

**Methods:**

Surveillance testing occurred every other week for non-vaccinated individuals and continued through February 2022. After May 2021, fully vaccinated individuals were tested monthly and encouraged to seek additional testing if symptoms or an exposure occurred. We assessed risk of positive test among faculty, student, and staff groups through a Cox proportional hazards regression, accounting for multiple events and time-dependent variables with the Andersen-Gill model. To account for inconsistent testing after vaccination, time was examined as number of tests rather than calendar time.

**Results:**

In total, 410 participants were followed during the observation period, with an average of 22 (SD 10.0, RNG 1-50) tests per person. A total of 9,452 tests were performed. There were 158 positive tests, with 60 (38%) occurring in January 2022 alone. When analyzed by themselves, staff and student groups were significantly more likely to test positive (HR 1.98, 95% CI 1.15-3.42; HR 2.16, 95% CI 1.29-3.63 respectively) compared to faculty. However, once additional covariates were accounted for, the relationship was no longer significant (Staff: HR 2.15, 95% CI 0.92-5.05; Students: HR 2.38, 95% CI 0.88-6.40).

Risk of COVID-19 within Dental School

**Figure:**
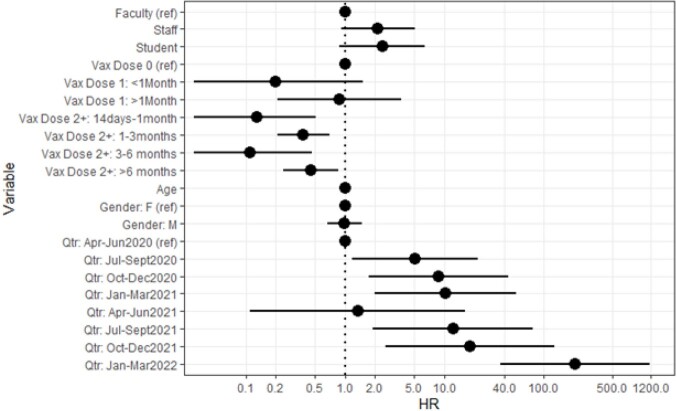

Hazard Ratios for testing positive for COVID-19 among different groups within the dental school. Vaccination is accounted for as time since last vaccine, with separate categories for one dose, and 2 or more doses combined. Time examined as test number.

**Conclusion:**

More than a third of all positive tests during the 22-month study occurred during one month of the Omicron wave. This sudden increase in positive tests was not observed in previous surges, and demonstrates the intensity of the Omicron wave. Additionally, we did not find a significant difference between patient-facing groups who had different work exposures. While this may be due to effective preventative measures, within the dental setting we do not see evidence that work role and resulting exposures increase risk.

**Disclosures:**

**All Authors**: No reported disclosures.

